# Ball heading and subclinical concussion in soccer as a risk factor for anterior cruciate ligament injury

**DOI:** 10.1186/s13018-021-02711-z

**Published:** 2021-09-19

**Authors:** George Kakavas, Nikolaos Malliaropoulos, Wieslaw Blach, Georgios Bikos, Filippo Migliorini, Nicola Maffulli

**Affiliations:** 1 Fysiotek Spine & Sports Lab, Athens, Greece; 2 Thessaloniki Sports Medicine Clinic, Thessaloniki, Greece; 3 Ports Clinic, Rheumatology Department, Barts Health NHS Trust, London, UK; 4 Centre for Sports and Exercise Medicine, Queen Mary, University of London, London, UK; 5 Faculty of Physical Education and Sport Sciences, University School of Physical Education, Paderewskiego 35, 51-612 Wroclaw, Poland; 6 European Judo Union, Vienna, Austria; 7 Euromedica-Arogi Rehabilitation Clinic, Pylaia, Thessaloniki, Greece; 8 Department of Orthopedic, Trauma, and Reconstructive Surgery, RWTH University Hospital, Pauwelsstrasse 31, 52064 Aachen, Germany; 9 London Sports Care, BMI London Independent Hospital, London, UK; 10 Department of Orthopaedics, School of Medicine, Surgery and Dentistry, University of Salerno, Salerno, Italy; 11 School of Pharmacy and Bioengineering, Keele University School of Medicine, Stoke on Trent, UK

**Keywords:** ACL, Concussion, Ball heading, Injury, Brain

## Abstract

Soccer players have a high risk of anterior cruciate ligament (ACL) injury, a potentially career-ending event. ACL rupture has been linked with abnormal neuromuscular control in the lower limb. Additionally, heading the ball with the unprotected head during game play is increasingly recognized as a major source of exposure to concussive and sub-concussive repetitive head impacts. This article provides a hypothesis of potential connection of ACL injury with ball heading in soccer players. The study reviews literature sources regarding the impact of neurocognitive alterations after ball headings in ACL injuries. Poor baseline neurocognitive performance or impairments in neurocognitive performance via sleep deprivation, psychological stress, or concussion can increase the risk for subsequent musculoskeletal injury.

## Introduction

In soccer, the most popular sport worldwide, players have a high risk of anterior cruciate ligament (ACL) injury, a potentially career-ending event [1]. There is a greater prevalence of ACL injuries in female soccer players, possibly because of biomechanical differences between males and females. Non-contact ACL injuries frequently occur during dribbling, cutting, or quick changes of direction, while contact injuries are caused by knee hyperextension or an abnormal valgus motion [2]. For these reasons, the biomechanics of knee joint motion during activities such as landing, running, and cutting maneuvers in soccer have been extensively studied.

The ACL ruptures when the stresses to which it is exposed exceed its mechanical properties, but extreme knee loading scenarios may be potentiated through abnormal neuromuscular control in the lower limb [3], with gender differences in hip rotation and rear foot pronation in the transverse and frontal planes [4].

Soccer accounts for a major number of sub-concussive episodes in sports [3]: excessive heading of the ball (more than 1000 episodes per year) may cause subclinical brain injury, the effects of which are not as well defined as those of recognized frank concussion. Although most published studies have focused on collegiate and professional players, most soccer players are amateur recreational league players [5].

Heading with the unprotected head to direct the ball during game play is increasingly recognized as a major source of exposure to concussive and sub-concussive repetitive head impacts [6]. These impacts have been linked to changes in brain structure visible on neuroimaging, and decreased performance on cognitive tasks both with short term [7] and long-term exposure [7–9].

Most studies on head injury in other sports have focused on single or repeated concussive injury episodes, now known to cause chronic brain pathology [10], especially in susceptible individuals. Recently, it was suggested that persistent effects on the central nervous system from soccer in adolescents were explained by collision-related injuries, not heading. However, as that investigation did not assess heading and its effects, it was not possible to assess the effects of unintentional vs intentional head impacts or exposures that occur during practice [11].

A recent imaging study [2] showed detectable structural differences in brain areas, consistent with traumatic brain injury (TBI), in amateur adult (mean age of 31 years, who played soccer since childhood) soccer players with self-reported high and low heading frequencies. Similar findings were also obtained in another recent imaging study [12] which evidenced significant differences in white matter integrity in a small sample of professional male soccer players (mean age of 20 years, who played soccer since childhood) compared with a control group of swimmers (mean age of 21 years).

## Ball heading as a subclinical concussion factor

Concussion involves several clinical domains: symptoms, physical signs, behavioral changes, cognitive impairment, and sleep disturbance. The physical signs of concussion can resolve quickly, but some players may manifest persistent impairment [13]. One study reported that intracortical inhibition within the primary motor cortex might be present more than 1 year after physical concussion symptoms have subsided. Impaired neural activity may affect several aspects of motor control, leading to decreased neuromechanical responsiveness. For example, decreased maximum strength has been reported after concussion [14].

Concussion can also result in decreased postural stability from impairment on the afferent signals from the cervical spine, the vestibular-ocular system, and the visual systems. Persistent sensorimotor impairment after resolution of concussion symptoms would likely contribute to an increased injury risk, and further studies are warranted. The long-term effects of repetitive cranial impacts from heading are an area of increasing concern, and exposure must be accurately measured; however, the validity of self-report of cumulative soccer heading is not known [15].

A study of NCAA (National Collegiate Athletic Association) Division I soccer players identified, with 74% sensitivity and 51% specificity, that lower neurocognitive reaction time was an indicator of elevated risk for lower extremity strains and sprains [12]. Alterations in kinematic and kinetic variables during movement have been extensively studied and identified as risk factors for ACL injury [11]. A recent observational cohort study showed that lower extremity stiffness is altered after a concussion in NCAA Division I soccer players: this could contribute to injury risk [4]. In addition, drop-jump landing biomechanics, a task frequently used to assess risk for ACL injury, vary with baseline neurocognition. Healthy athletes without concussion but lower neurocognitive performance demonstrated movement patterns associated with increased risk of ACL injury. Thus, based on the evidence presented of higher risk of musculoskeletal injuries after concussion, screening for neuromuscular risk factors associated with lower extremity injury should be incorporated into return to sport protocols following concussion [16].

When considering the aspects of neurocognition most likely related to athletic performance, the relationship between neurocognition and neuromuscular performance may be mediated by visual attention, self-monitoring, fine motor performance, reaction time, and dual tasking [14]. These neurocognitive dimensions are likely critical for the performance and safety of athletes as they attempt to initiate and maintain appropriate neuromuscular responses to the demands of dynamic activities in a constantly evolving competitive environment. For example, quick reaction times may allow athletes to rapidly adjust to new demands during play while maximizing performance. Conversely, poor reaction times may reduce athletes’ ability to react to evolving demands during competition in an adequate time frame to allow for a safe and appropriate response [17]. Similarly, athletes who are poor at dual tasking may not be able to appropriately monitor the neuromuscular performance associated with the athletic task while devoting visual attention to an opponent. Thus, athletes with low neurocognitive performance may be hindered in their ability to plan, time, perform, and/or monitor neuromuscular performance during athletic tasks.

These neurocognitive dimensions are likely highly intertwined with neuromuscular control, motor learning, and other aspects critical for the performance and safety of the athlete. Sport activities demand initiating and maintaining appropriate performance of dynamic activities in a complex, rapidly changing environment. The success of each action is contingent on voluntary and involuntary motor commands modulated by sensory processing, attention, and motor planning. Appropriate function in these neurocognitive dimensions would allow athletes to accomplish motor tasks successfully and safely.

In the present work, we have focused on the possible connection of subclinical head injury and impairment in proprioception as a possible causative factor in ACL injury. We are however aware of the large body of evidence on concussion in American football players, which shows that concussed athletes have increased odds of sustaining an acute lower extremity musculoskeletal injury after return to play than their nonconcussed teammates. The study results suggest further investigation of neurocognitive and motor control deficits may be warranted beyond the acute injury phase to decrease risk for subsequent injury. The topic is extremely vast, and in this respect, ACL injury is only a relatively minor issue to tackle. Head injury prevention programs span well beyond ACL injury, and their impact will extend to prevention of impairment of neural function and neurocognition.

From a sports traumatology and rehabilitation perspective, we should try and produce intervention models first which allow to assess neurocognitive performance and identify athletes at risk for injury. Also, in the rehabilitation process, neuromuscular training tools should incorporate progressively more challenging tasks. The benefits of using tasks such as dual-attention during clinical assessment are currently being explored when assessing and managing concussion [17]. This strategy can be successfully translated to ACL injury risk screening, and neurocognitive strategies may be employed in ACL injury prevention and ACL injury rehabilitation [18]. Given the relationship between neurocognition and neuromuscular performance described above, prevention and rehabilitation efforts should also incorporate some aspects of neurocognition. Athletes at more advanced stages of a given injury prevention or rehabilitation program should practice tasks with neurocognitive challenges such as dual tasking, visual attention, and reaction time among others [5].

## Conclusion

Neuroscience will continue to help uncover how the nervous system influences and determines motor control, and the mechanistic errors in motor control resulting in non-contact ACL injury. Poor baseline neurocognitive performance or impairments in neurocognitive performance via sleep deprivation, psychological stress, or concussion injury can increase the risk for subsequent musculoskeletal injury. Knowledge of the relationship between neurocognitive performance and musculoskeletal injury risk could supplement the current neuromuscular-focused paradigm of injury risk screening, rehabilitation, and prevention.

## Data Availability

This study does not contain any third material.

## Abbreviations

TBI: Traumatic brain injury
NCAA: National Collegiate Athletic Association
ACL: Anterior cruciate ligament
